# Identification of Optimum Sequencing Depth Especially for *De Novo* Genome Assembly of Small Genomes Using Next Generation Sequencing Data

**DOI:** 10.1371/journal.pone.0060204

**Published:** 2013-04-12

**Authors:** Aarti Desai, Veer Singh Marwah, Akshay Yadav, Vineet Jha, Kishor Dhaygude, Ujwala Bangar, Vivek Kulkarni, Abhay Jere

**Affiliations:** Persistent LABS, Persistent Systems Ltd., Pune, Maharashtra, India; Queen's University Belfast, United Kingdom

## Abstract

Next Generation Sequencing (NGS) is a disruptive technology that has found widespread acceptance in the life sciences research community. The high throughput and low cost of sequencing has encouraged researchers to undertake ambitious genomic projects, especially in *de novo* genome sequencing. Currently, NGS systems generate sequence data as short reads and d*e novo* genome assembly using these short reads is computationally very intensive. Due to lower cost of sequencing and higher throughput, NGS systems now provide the ability to sequence genomes at high depth. However, currently no report is available highlighting the impact of high sequence depth on genome assembly using real data sets and multiple assembly algorithms. Recently, some studies have evaluated the impact of sequence coverage, error rate and average read length on genome assembly using multiple assembly algorithms, however, these evaluations were performed using simulated datasets. One limitation of using simulated datasets is that variables such as error rates, read length and coverage which are known to impact genome assembly are carefully controlled. Hence, this study was undertaken to identify the minimum depth of sequencing required for *de novo* assembly for different sized genomes using graph based assembly algorithms and real datasets. Illumina reads for *E.coli* (4.6 MB) *S.kudriavzevii* (11.18 MB) and *C.elegans* (100 MB) were assembled using SOAPdenovo, Velvet, ABySS, Meraculous and IDBA-UD. Our analysis shows that 50X is the optimum read depth for assembling these genomes using all assemblers except Meraculous which requires 100X read depth. Moreover, our analysis shows that *de novo* assembly from 50X read data requires only 6–40 GB RAM depending on the genome size and assembly algorithm used. We believe that this information can be extremely valuable for researchers in designing experiments and multiplexing which will enable optimum utilization of sequencing as well as analysis resources.

## Introduction

Traditionally, Sanger sequencing was used to sequence the genomes of organisms of interest. Due to the cost and technology limitations in generating the sequence data, genomes were sequenced at approximately 6–10X coverage in order to generate draft genome assemblies [1]; [2]. Using Sanger sequencing technology, the human genome was sequenced at 6–8X average coverage and cost of about $ 2.7 billion and required efforts from over 3000 scientists from 6 different countries [3]. The complexity, cost and time involved in the human genome project, highlighted the dire need for the development of sequencers with higher throughput and lower cost of sequencing. This need culminated in the development of multiple high throughput or massively parallel sequencing technologies collectively referred to as the Next Generation Sequencing (NGS) technologies. Current NGS systems generate data in the form of millions of short (75–300 bp) reads [4]; [5] and produce data ranging from 1****GB to 600****GB depending on the sequencing platform used. Additionally, the cost of sequencing on NGS systems is much lower as compared to the automated Sanger sequencing method. According to the data released by the National Human Genome Research Institute, the cost of sequencing a human sized genome using the NGS technology is a little less than $10000 and this includes library preparation, sequencing and data analysis (http://www.genome.gov/sequencingcosts/).

As a result, NGS systems have drastically accelerated the research involving large scale sequencing and globally researchers have now undertaken large sequencing projects involving *de novo* genome assembly [6]; [7]; [8] and metagenomic studies [9]; [10] for new species, resequencing [11], exome sequencing [12]; [13]; [14], transcriptome profiling [15]; [16] and methylation profiling [17]; [18]; [19] for known genomes. Since NGS technologies produce sequence as short reads and have a higher error rate (0.1–1% depending on the sequencing platform) [20]; [21], higher depth of sequencing is recommended. Moreover using current NGS platforms, 60X to 100X coverage for large genomes and a few 100X coverage for small genomes can be easily achieved. However, sequence generation is only one aspect of sequencing projects and the analysis of large quantum of data generated by NGS platforms, presents a significant bioinformatics and computational challenge, particularly for *de novo* genome assembly (which involves generation of a novel previously unknown sequence entirely from the available sequence read data). The outcome of performing *de novo* genome assembly is a draft “reference sequence” of the genome of the organism of interest and availability of reference sequence for a variety of organisms by *de novo* assembly is important for the advancement of genomic research.

Higher read depth for NGS (as compared to Sanger sequencing) seems to be a prerequisite, but there have been no systematic efforts to identify the optimum read depth that would be sufficient to assemble a genome. This is an important question to answer as *de novo* assembly of millions of short reads is a computationally and bioinformatically challenging task. Recently, some studies have evaluated the impact of sequence coverage, error rate and average read length on genome assembly [22] as well as compared the currently available assembly tools [23]; [24], however, these evaluations were performed using simulated datasets and not using real data sets. One obvious limitation of using simulated datasets is that, it presents a best case scenario for analysis where variables such as coverage, error rates and read length that are known to impact genome assembly are carefully controlled. On the other hand, assembly using real datasets is complicated by factors such as non-uniformity of coverage [25], variable error rates (with higher errors towards the end of the read) [20] and variable read length [26]; [21].

In the present study, we attempt to identify the optimum average depth required to generate a “good” whole genome assembly using five De bruijn graph based short reads assemblers; SOAPdenovo [27], Velvet [28], Meraculous [29] IDBA-UD [30] and ABySS [31]. Even though we have selected these five assemblers for this study, there are several other excellent assembly algorithms such as ALLPaths LG [32], Celera [33], RAY [34], SSAKE [35] and VCAKE [36] that are available for assembling genomes. The five algorithms we have used in this study were selected because Velvet, SOAPdenovo and ABySS are widely used, whereas Meraculous and IDBA-UD are recently published and claim to outperform the previously published algorithms. The sequence data used in this study was from Illumina GA at an average depth of 990X for *E.coli*, 275X for *S.kudriavzevii* [37] and 200X for *C.elegans* [38]. We focused on Illumina data as it is the most widely used platform for generating sequence data and its high throughput enables deep sequencing of genomes, particularly small genomes. We chose the De bruijn graph based algorithms as they are best suited for assembling short reads generated by majority of the current NGS systems [39]. Commonly, De bruijn graph based assembly algorithms break reads into k-mers of specified length as originally proposed by Pevzner [40] and overlapping k-mers are identified as the nodes of the graph and a directed edge between nodes indicates that the k-mers of these nodes occur sequentially in one or more reads. Stretches of DNA sequence with non-ambiguous bases form non-branching paths in the De bruijn graph and these can be easily converted into contigs by reading along the path.

Our results suggest that, considering metrics such as genome coverage, N50 (the length of the smallest contig which when added to a set of larger contigs yields at least 50% of the genome) [41], maximum contig size, number of contigs and errors in assembly, read depth of 50X is enough to get a “good” genome assembly and sequencing at a depth greater than 100X does not provide any additional benefits. Additionally, we observed that computational resources required for assembling read data of 20 X–100 X depth is between 6–40****GB, for small genomes of *E.coli* and *S.kudriavzevii*.

## Methods

### Assembly algorithms

Five graph-based short read assemblers; SOAPdenovo (Release 1.05, 14-02-2011) [27], Velvet [28], Meraculous [29] IDBA-UD [30] and ABySS [31] were selected for this study. All these assemblers are based on De bruijn graph approach. They are publically available and are widely used for *de novo* assembly of short reads generated by NGS platforms such as Illumina Genome Analyzer, HiSeq and SOLiD. All of them support assembly using paired end data and were run with default parameters.

All the genome assembly work was carried out using a dual Quad-core (2.4 GHz) Linux server with 128 GB RAM.

### Data Sets

The short read data for *E.coli* strain MG1655 (ERR022075; Read length: 2X100 bp and Insert size: 311 bp), *S.kudriavzevii* (SRR173086; Read length: 2 X 114 bp and Insert size: 226±23 bp) and *C.elegans* (SRR065388; Insert size: 206 bp and SRR065390; Insert size: 356 bp. Read length for both data set: 2X100 bp) was obtained from Sequence Read Archive (SRA), NCBI. The data was generated on Illumina Genome Analyzer from paired end libraries and have read depth of 990X, 275X and 200X respectively.

### Sub Sizing the Data

The data downloaded from SRA had depth of coverage of 990X for ERR022075 (*E.coli*), 275X for SRR173086 (*S.kudriavzevii)* and 200X for SRR065388 and SRR065390 (*C.elegans)*. Since, our aim was to identify optimum read depth to produce a good quality genome assembly, we randomly sub-sized the datasets. Perl script was written for randomly sub-sizing the data to generate a range of depth of coverage; 20X, 35X, 50X, 100X, 150X and 200X. We performed assembly for each of the sub-sized blocks within each of the datasets and did not find significant difference between the assembled genomes (data not shown). This indicates that sub-sizing the data had no impact on the final outcome.

### Metrics for accessing the quality of *de novo* assembled genome

For each of the depth and each of the assemblers, we used genome coverage (after comparing with the reference genome) and N50 values as the deciding criteria for identifying a “good” assembly. The metrics were obtained on scaffolds generated by the respective assemblers. The depth of coverage that provided the best genome coverage and N50 was identified as the optimum depth of sequencing. Genome coverage is an especially useful metric if the reference sequence of the organism itself or a relative is known. Additional metrics such as number of contigs and average contig size were also tracked.

#### Genome coverage

The assembly validation script designed for the GAGE (Genome Assembly Gold-Standard Evaluations) [42] assembly comparison project was used to evaluate the validity of the assemblies produced in this study. The assembly validation scripts make use of MUMmer [43] alignment tool to compare the assembled contigs to the reference genome. MUMmer is a modular system for fast whole genome alignment of finished or draft genome sequences. It can easily handle hundreds to thousands of contigs; align them to another set of contigs or a genome using the NUCmer program [43]. For alignment with NUCmer, the default parameters used were; Minimum match –20, % identify –95 and % coverage –95.

For *E.coli*, the reference genome used was NC_000913 and for *S.kudriavzevii* the reference genome used was ZP_591. The *C.elegans* reference genome was NC_003279.7, NC_003280.0, NC_003281.9, NC_003282.7, NC_003283.10 and NC_003284.8 whereas the mitochondrial genome used was NC_001328.1

#### N50, Number of contigs and Average contig size

The N50 (the length of the smallest contig which when added to a set of larger contigs yields at least 50% of the genome) value is widely used for assembly algorithm comparison [23]; [24] and higher the N50 value better the assembly, provided high genome coverage is achieved. Number of contigs and average contig size give an estimate of the size of the pieces that make up the assembly. Therefore small number of contigs and high average contig size are indicators of a good genome assembly.

#### Accuracy of the assembly

The GAGE script that was used to identify genome coverage also generates metrics such as number of SNPs and InDels that can be used to evaluate the accuracy of the assembled genome. Presence of high number of SNPs in the assembled genome would indicate an incorrect consensus base incorporated in the assembled genome.

## Results and Discussion

There has been an increase in the number of *de novo* genome assemblies generated using NGS data [8]; [27] as well as the number of assemblers available to assemble this data [27]; [28]; [32]. All of the short read assemblers are based on De bruijn graph approach and a number of studies evaluating the performance of these assembly algorithms for genomes of different sizes have been published in recent years [22]; [23]; [24]. However, majority of these studies were conducted using simulated data sets with defined error rates and uniform depth of coverage. Moreover, these studies were focused on metrics such as time required for genome assembly, computational resources utilized and effect of read length, genome size and error rate on genome assembly. With increasing throughput of NGS systems, it is important to understand how much sequencing is needed for “good” genome assembly, so as to minimize the cost of sequencing per sample while fully utilizing the potential of the sequencers through multiplexing. Additionally, the sequencing chemistry of NGS systems is continuously improving thus reducing the error rate; hence it may not be necessary to sequence DNA at a very high depth.

The objective of this study was to identify the optimal sequencing depth required to assemble small to mid-sized genomes (up to 100 MB) using some of the most commonly used as well as recently published assemblers. We obtained data generated on Illumina Genome Analyzer for three genomes of varying size; *E.coli* MG1655 (4.63 MB), *S.kudriavzevii* (11.18 MB) and *C.elegans* (100 MB) at 990X, 275X and 200X respectively, randomly sub-sized the data and assembled them using five graph based assembly algorithms; Velvet, SOAPdenovo, Meraculous, ABySS and IDBA-UD. We used the GAGE scripts to compare the assembled genomes with the reference genome to identify the genome coverage. In some cases, the genome coverage was greater than 100%, which we believe could be because of the presence of repeat region in the genome. Importantly, we were able to generate good genome assembly (95% or greater genome covered after comparative analysis) with Velvet, SOAPdenovo, ABySS and IDBA-UD (not in case of *C.elegans*), but not Meraculous, with as low as 20X depth of coverage (**Tables 1** **,** **2** **and** **3** **; See below for detailed discussion**). This is the first time that it has been shown, using experimental data that such low coverage can yield an acceptable genome assembly for small organisms such as bacteria, yeast and worm. At the same time, we have demonstrated that increasing the depth of sequencing does not provide significant advantage when using Velvet, ABySS, SOAPdenovo and IDBA-UD but increasing the depth of sequencing significantly improves the assembly generated by Meraculous. However, even in the case of Meraculous, sequencing depth >100X does not provide a significant benefit for small genomes such as *E.coli* and *S.kudriavzevii*. Additionally, computational resources required for assembling read data of 20 X–100 X depth ranges from 6–40 GB, for small genomes depending on the assembler used.

**Table 1:** Assembly metrics for *E.coli* genome assembled from Illumina paired end data with Velvet, SOAPdenovo, ABySS, Meraculous and IDBA-UD.

	Velvet		SOAPdenovo	ABySS		Meraculous	IDBA-UD
Number of Contigs							
20X	284		280	448		1510	215
35X	251		198	278		362	202
50X	209		253	185		292	233
100X	157		279	153		148	258
150X	120		267	146		124	305
200X	158		311	146		115	289
Average Contig Length							
20X	16156.8		16231.5	10202.9		1419.23	21189.7
35X	18221.6		23034.6	16627.7		11939.2	22628.4
50X	21895		18078.4	25156.3		15031.7	19647.6
100X	29133.9		16432.1	30395.1		30373.8	17780.1
150X	38100.8		17176.9	31889.4		36368.4	15067.2
200X	28964.4		14778.4	31921.8		39274.2	15895.1
Maximum Contig Length							
20X	178348		221554	100991		13023	173604
35X	221572		314900	222650		74711	203084
50X	284242		268507	222853		125799	221677
100X	293696		312097	222915		178290	224008
150X	221612		221639	222915		178333	268172
200X	242677		315391	222915		221617	224008
Genome coverage (bases)							
20X	4505595 (97.11%)	4521190 (97.44%)			4556108 (98.19%)	2008137 (43.28%)	4554438 (98.16%)
35X	4600283 (99.15%)	4549085 (98.04%)			4613734 (99.44%)	4294176 (92.55%)	4570413 (98.5%)
50X	4561865 (98.332%)	4561639 (98.31%)			4649731 (100.21%)	4368129 (94.14%)	4577197 (98.65%)
100X	4567690 (98.44%)	4576959 (98.64%)			4649792 (100.21%)	4482341 (96.6%)	4586801 (98.86%)
150X	4567362 (98.44%)	4582652 (98.77%)			4655357 (100.33%)	4500522 (97%)	4594933 (99.03%)
200X	4570779 (98.51%)	4592154 (98.97%)			4660225 (100.44%)	4509122 (97.18%)	4593102 (98.99%)

Error in assembled genome										
	SNP	InDel	SNP	InDel	SNP	InDel	SNP	InDel	SNP	InDel
20X	91	28	90	78	107	475	1	457	119	32
35X	361	83	109	40	148	102	2	159	79	40
50X	113	11	76	24	87	43	2	127	45	66
100X	117	27	37	91	83	33	2	56	19	42
150X	83	24	54	108	64	30	2	42	14	67
200X	123	43	110	79	107	32	2	19	15	58

The expected genome size for *E.coli* MG1655 is 4639675 bases.

**Table 2:** Assembly metrics for *S.kudriavzevii* genome assembled from Illumina paired end data with Velvet, SOAPdenovo, ABySS, Meraculous and IDBA-UD.

	Velvet	SOAPdenovo	ABySS	Meraculous	IDBA-UD
Number of Contigs					
20X	3647	3421	5524	8323	1203
35X	1424	1167	3080	2414	1773
50X	661	2145	2243	2097	1879
100X	524	3102	1916	744	1991
150X	573	3763	1850	594	1960
200X	711	4689	2200	573	2012
Average Contig Length (bases)					
20X	3221.18	3377.79	2103.14	328.911	9674.6
35X	8177.24	10267.7	3828.97	4717.08	6624.67
50X	17617.6	5472.39	5269.26	5386.86	6262.4
100X	22199.2	3838.7	6180.79	15417.2	5929.76
150X	20328.5	3198.61	6415.42	19336.2	6024.7
200X	16432.7	2599.94	5407.71	20063.2	5875.02
Maximum Contig Length (bases)					
20X	32734	47695	27667	5483	215790
35X	320896	220365	175589	48857	215367
50X	207865	478251	129807	60475	188478
100X	396838	377320	391375	175552	329736
150X	404654	458019	391364	360534	348439
200X	381309	348264	350371	293610	348443
Genome Coverage (bases)					
20X	11137714 (99.42%)	11099527 (99.08%)	11149205 (99.53%0	2613522 (23.33%)	11148577 (99.52%)
35X	11221514 (100.17%)	11146583 (99.50%)	11227483 (100.23%)	11123809 (99.30%)	11268339 (100.59%)
50X	11176252 (99.77%)	11219577 (100.15%)	11245724 (100.39%)	11002676 (98.22%)	11271642 (100.62%)
100X	11200115 (99.98%)	11260111 (100.52%)	11236212 (100.30%)	11167029 (99.69%)	11273408 (100.64%)
150X	11207895 (100.05%)	11292045 (100.80%)	11237111 (100.31%)	11180363 (99.80%)	11274406 (100.64%)
200X	11222467 (110.18%)	11339559 (101.23%)	11240746 (100.34%)	11182471 (99.82%)	11274323 (100.64%)

Error in assembled genome										
	SNP	InDel	SNP	InDel	SNP	InDel	SNP	InDel	SNP	InDel
20X	441	329	343	2611	159	292	58	62	423	511
35X	243	751	181	232	580	491	46	199	235	482
50X	250	342	177	1267	179	453	84	457	215	446
100X	170	443	155	1566	349	539	71	300	209	485
150X	189	485	224	1773	271	505	93	324	236	502
200X	161	564	292	1778	348	506	75	298	215	497

The size of the assembled genome we used ZP_591 as reference is 11201698 bases.

**Table 3:** Assembly metrics for *C.elegans* genome assembled from Illumina paired end data with Velvet, SOAPdenovo, ABySS, Meraculous and IDBA-UD.

	Velvet	SOAPdenovo	ABySS	Meraculous	IDBA-UD
Number of Contigs					
20X	64450	124333	158419	127344	45380
35X	50223	54175	130592		26174
50X	20369	58430	113521	5130	26049
100X	12852	54936	83051	76890	31682
150X	10362	55090	73487	40161	41191
200X	9547	56810	70275	25002	49908
Average Contig size					
20X	1581.05	805.639	656.37	201.977	2090.02
35X	1988.3	1842.2	803.407		3837.33
50X	4983.68	1756.63	940.162	335.264	3855.73
100X	7928.07	1897.26	1298.32	644.795	3240.16
150X	9863.32	1907.95	1476.72	2445.47	2529.5
200X	10715	1860.29	1549.11	3692.64	2112
Maximum Contig length					
20X	39794	27376	35861	11562	58626
35X	90829	135554	102365		154577
50X	395204	115246	125515	13606	232754
100X	406962	116690	192510	15948	270543
150X	403179	142926	203282	125692	200042
200X	442196	120281	384612	86439	270543
Genome Coverage (bases)					
20X	95125613 (94.85%)	95426999 (95.15%)	96843084 (96.57%)	24649177 (24.58%)	90021226 (89.76%)
35X	94709449 (94.44%)	94868880 (94.60%)	97717867 (97.44%)	Data Not Generated	95559209 (95.29%)
50X	95784402 (95.51%)	97746194 (97.47%)	99358917 (99.08%)	1620321 (1.61%)	95548487 (95.28%)
100X	96294494 (96.02%)	99077027 (98.79%)	10133828 (101.05%)3	48129645 (47.99%)	97789517 (97.51%)
150X	96576131 (96.35%)	99904107 (99.62%)	103018960 (102.02%)	93574066 (93.31%)	99334562 (99.05%)
200X	96671928 (96.40%)	100428367 (100.14%)	103378677 (103.08%)	88472503 (88.22%)	100530260 (100.2%)

Error in assembled genome										
	SNP	InDel	SNP	InDel	SNP	InDel	SNP	InDel	SNP	InDel
20X	11853	4865	11704	3015	5490	2725	751	436	23089	5649
35X	10536	6384	5619	3002	5497	2758	Data Not Generated		15565	4947
50X	12491	7423	6326	2748	8675	3497	46	42	14865	5082
100X	11205	6062	5900	2838	8071	4148	725	4975	10721	4474
150X	11026	5303	5065	2777	8107	4505	1650	2692	8787	4238
200X	11801	5214	5119	2767	8152	4902	1093	6126	7616	4067

The expected genome size of the *C.elegans* genome is 100281427.

### E.coli MG1655 de novo assembly

**Figure 1:**
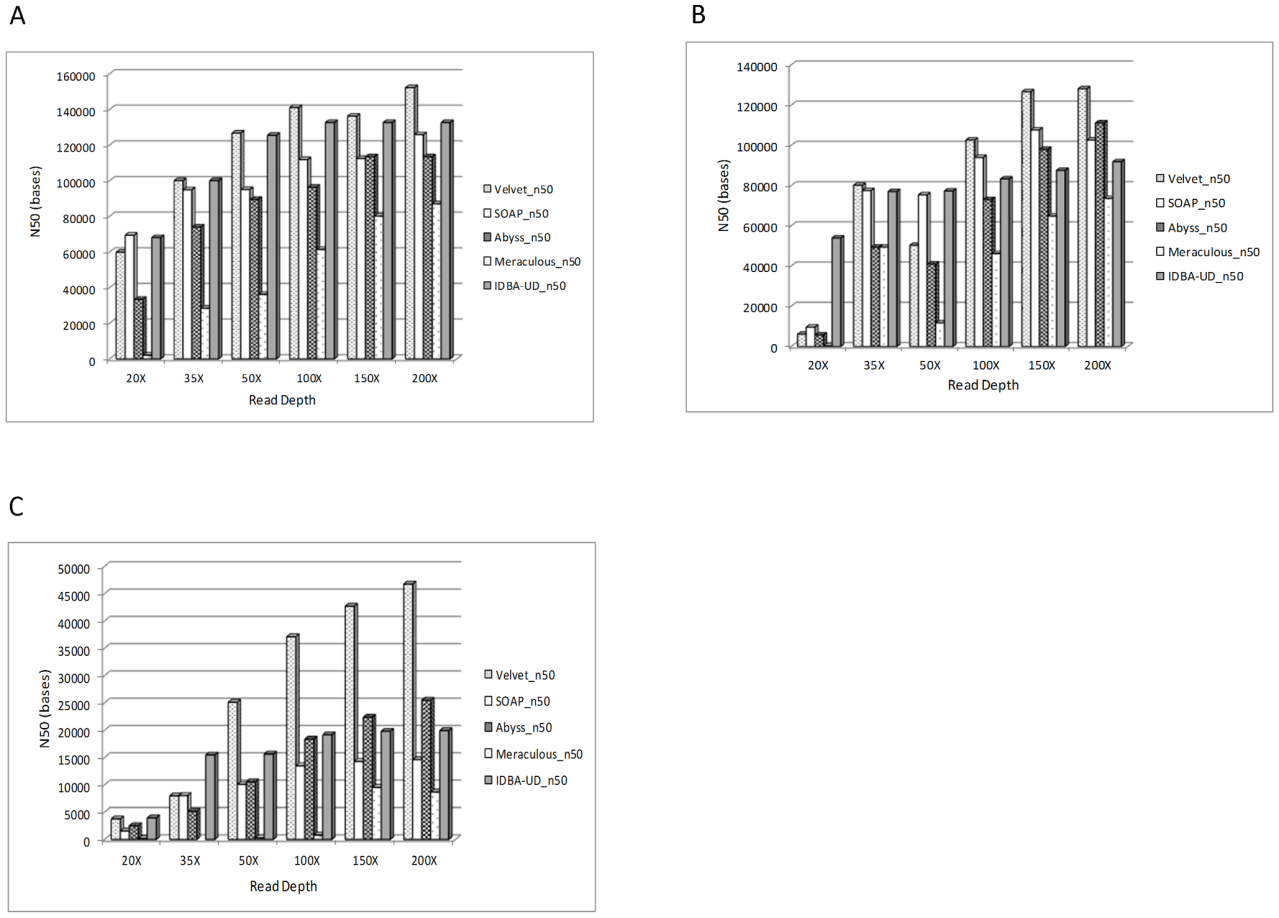
N50 value for the genomes assembled by Velvet, SOAPdenovo, ABySS, Meraculous and IDBA-UD. **A**) N50 for assembled *E.coli* genome: N50 is the length of the smallest contig which when added to a set of larger contigs yields at least 50% of the genome. The N50 values for IDBA-UD, Velvet and SOAPdenovo seemed to reach a plateau at 35X, ABySS at 50X depth of coverage. On the other hand, the N50 value of Meraculous generated assembly increased till 150X depth of coverage. **B**) N50 for assembled *S.kudriavzevii* genome: IDBA-UD and SOAPdenovo attained peak N50 value at 35X and 100X depth of coverage respectively, whereas the N50 value of Velvet, ABySS and Meraculous generated assembly increased till 150X depth of coverage. **C**) N50 for assembled *C.elegans* genome: SOAPdenovo, ABySS and IDBA-UD reached peak N50 value at 100X depth of coverage, whereas the N50 value of Velvet generated assembly increased approximately 1.5 fold until 150X with no change thereafter. Velvet generated assembly had the best N50 values of all the 4 assemblers.

*E.coli* Illumina reads at 20X, 35X, 50X, 100X, 150X and 200X were assembled using SOAPdenovo, Velvet, ABySS, Meraculous and IDBA-UD. The De bruijn graph based algorithm use a subset of read called k-mer in order to identify overlaps between reads. The k-mer value has a significant impact on the final assembly, hence reads at each read depth were assembled using a range of k-mer (55–91 for 50X–100X and 31–97 for 35X with steps of 2) to identify the optimal k-mer for a given read depth (Data not shown). K-mer corresponding to the maximum genome coverage was taken as the optimum k-mer. The % genome covered (after comparing with the reference genome) and N50 at the best performing k-mer at each read depth was used to identify the optimum read depth for assembling *E.coli* genome. Our analysis reveals that in the case of this particular dataset, with as low as 20X, we could achieve >97% coverage with all assemblers, except for Meraculous (**Table 1**). The N50 value also did not change significantly from 35X to 200X for all assemblers except for Meraculous where there is an approximately 2 fold increase in the N50 value from 35X to 100X and approximately 3 fold increase from 35X to 150X and 200X (**Fig. 1A**). Moreover, when additional metrics such as average contig size, number of contigs, maximum contig length and number of SNPs and InDels (**Table 1**) in the assembled genome are considered, 50X read depth appears to be optimum read depth for assembling *E.coli* genome using all assemblers except for Meraculous. In case of Meraculous, there is a 2 fold decrease in the number of contigs and a 2 fold increase in the average contig length as well as a 1.5 fold increase in the maximum contig length when the read depth is increased from 50X to 100X with no significant increase in these metric thereafter (**Table 1**). Moreover, in case of Meraculous there is a 2 fold decrease in the number of InDels present in the assembled genome from 50X to 100X (**Table 1**). On comparing genome assembled using Velvet, SOAPdenovo, ABySS and IDBA-UD with the known reference (SNPs and InDels generated by the GAGE script) there was a decrease in SNPs when the sequencing depth increased to 150X, with the number of SNPs actually increasing at 200X read depth (**Table 1**). Assembly generated by Meraculous has only 1-2 SNPs at all of the sequencing depth (**Table 1**).

**Figure 2:**
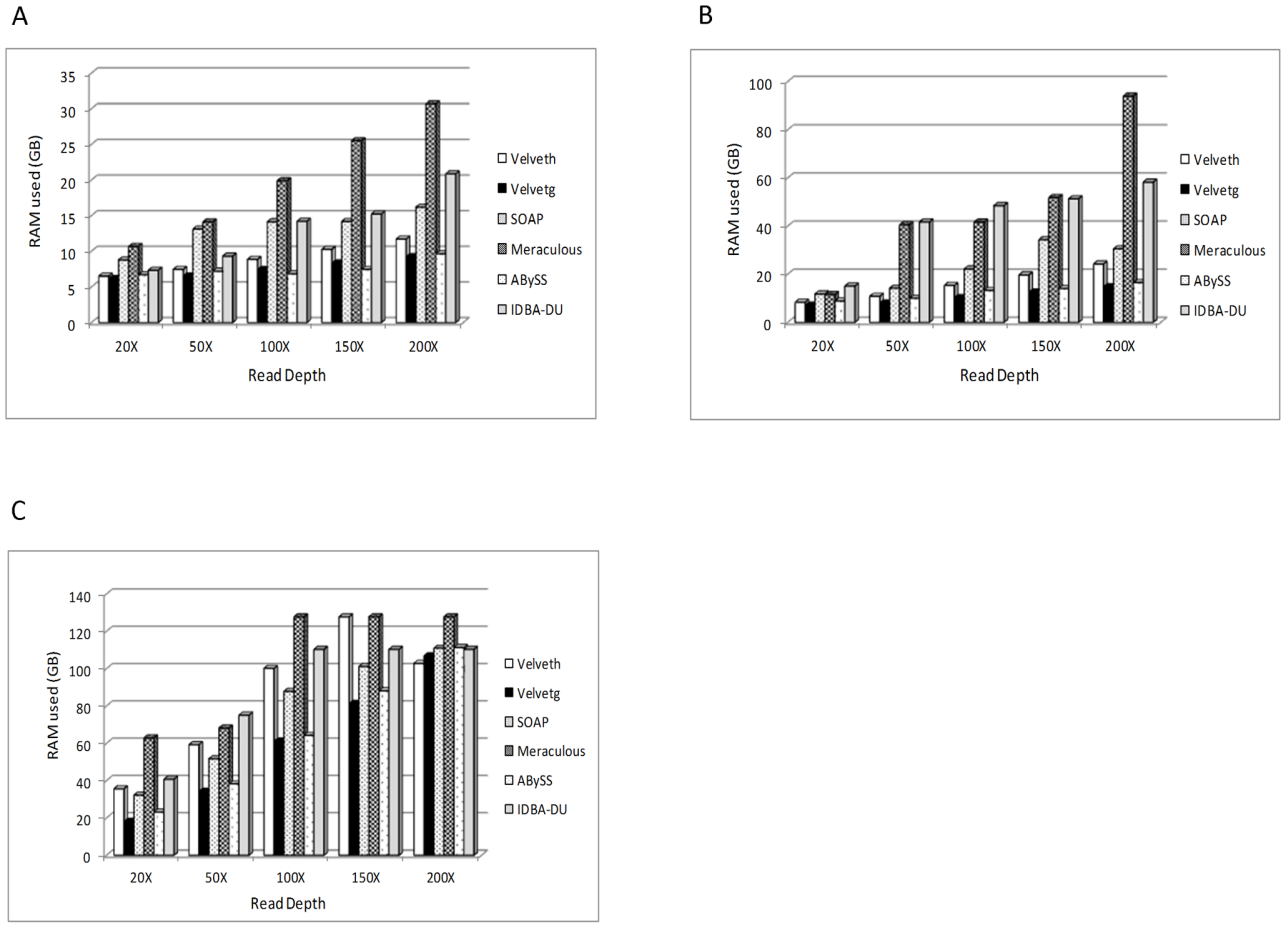
Memory requirement for genome assembly. Memory required to assemble *E.coli* (**A**), *S.kudriavzevii* (**B**) and *C.elegans* (**C**) genomes increased, although not proportionately, with increasing depth of sequencing.

We also measured the memory requirements for assembling read data at different depth and found that the read data of up to 50X depth can be assembled with 6–16 GB RAM depending on the assembly algorithm (**Fig. 2A**). Velvet and ABySS were the most memory efficient in assembling the *E. coli* genome followed by SOAPdenovo. Meraculous was the least memory efficient assembly algorithm. Thus, for assembling *E.coli* genome using Illumina sequence data, 50X coverage was sufficient to provide >98.5% coverage of the genome using Velvet, SOAPdenovo, ABySS and IDBA-UD, whereas to achieve same coverage a sequencing depth of 100X was required when using Meraculous.

### *S.kudriavzevii de novo* assembly

For *S.kudriavzevii,* Illumina reads at 20X, 35X, 50X, 100X, 150X and 200X were assembled using SOAPdenovo, Velvet, ABySS, Meraculous and IDBA-UD. Reads at each read depth were assembled using a range of k-mer (55–91 for 50X–100X and 31–97 for 35X with steps of 2) to identify the optimal k-mer for a given read depth. Our analysis revealed that for this dataset, with as low as 20X, we could achieve >95% coverage with all assemblers, except for Meraculous (**Table 2**). Genome coverage of greater than 99% was obtained for Velvet, SOAPdenovo, ABySS and IDBA for as low as 35X coverage and there was a negligible increase in genome coverage with increasing sequencing depth (**Table 2**). The N50 value however painted a slightly different picture. The N50 value increased gradually for all assemblers (except IDBA-UD) from 35X to 100X with non-significant increase thereafter (**Fig. 1B**). The N50 value for assemblies generated by IDBA-UD seems to plateau at 35X depth of coverage. The maximum contig length and average contig size did not change substantially from 35X to 200X depth of coverage for Velvet, SOAPdenovo, ABySS and IDBA-UD, but the number of contigs decreased by 2 fold only for Velvet. Additionally, for Meraculous, there was a 3 fold decrease in the number of contigs and a 3 fold increase in both, average contig length and maximum contig length when depth was increased from 50X to 100X (**Table 2**). We also evaluated the accuracy of the assembled genomes by analyzing the number of SNPs and InDels found in the genomes. There was no consistent pattern in the number of SNPs and InDels found in the assembled genomes across all the different depth and the assemblers (**Table 2**). Measurement of computational requirements showed that read data of up to 100X depth can be assembled with 12–40 GB RAM depending on the assembly algorithm (**Fig. 2B**). ABySS was the most memory efficient in assembling the yeast genome followed by Velvet. Meraculous was again the least memory efficient assembly algorithm requiring as much as 96 GB RAM to assemble the *S.kudriavzevii* genome from the 200X dataset.

### *C.elegans de novo* genome assembly

For *C.elegans,* Illumina reads at 20X, 35X, 50X, 100X, 150X and 200X were assembled using SOAPdenovo, Velvet, ABySS, Meraculous and IDBA-UD. Reads at each read depth were assembled using a range of k-mer (55–91 for 50X–100X and 35–95 for 35X with steps of 2) to identify the optimal k-mer for a given read depth. Our analysis revealed that for the dataset used in this study, we could achieve approximately 95% coverage with Velvet, SOAPdenovo and ABySS, but only 24% with Meraculous and approximately 90% with IDBA-UD (**Table 3**). Meraculous seems to be particularly poor at assembling moderate sized genome as the genome coverage achieved at even at 150X depth was 93% (**Table 3**). Due to this inconsistent performance, we did not generate *C.elegans* genome assembly using data with 35X read depth. Velvet, SOAPdenovo, ABySS and IDBA-UD were able to generate genome with approximately 95% coverage with as low as 35X read depth (**Table 3**). The N50 value increased by approximately 3 fold and 1.8 fold for Velvet and ABySS respectively, from 35X to 50X with gradual increase thereafter. On analyzing the SNP and InDel data, we observed that Velvet generated assembly had the highest number of SNPs and InDels, whereas ABySS produced assemblies with least number of errors (**Table 3**). Measurement of computational requirements showed that assembly of *C.elegans* genome requires a large amount of RAM at all the read depths. Approximately 24 GB, 32 GB, 36 GB, 40GB and 62 GB RAM is required by ABySS, SOAPdenovo, Velvet, IDBA-UD and Meraculous respectively for 20X read data. On the other hand, approximately 110 GB RAM is required by ABySS, SOAPdenovo, Velvet and IDBA-UD, whereas 128 GB RAM was required by Meraculous to assemble *C.elegans* genome from 200X read data (**Fig. 2C**). Thus, for assembling *C.elegans* genome using Illumina sequence data, 50X depth was sufficient to provide approximately 99% coverage of genome using ABySS, 100X depth was sufficient to provide approximately coverage of the genome using SOAPdenovo, whereas with the data set used in this study, Meraculous was able to achieve only 93% genome coverage with 150X read depth.

## Conclusions

Unlike large mammalian genomes where the depth of coverage achieved is typically low (between 20X and 60X) [8]; [27] due to the small size, genomes of small organisms get sequenced at a much higher depth. Sequencing at higher depth without any benefit to the outcome is wasteful not only from computational resource perspective, but also of sequencing resources perspective.

Our results show that 35X–50X data obtained from NGS platforms such as Illumina is sufficient to get good coverage of small genomes such as bacteria and yeast. For moderate sized genome such as *C.elegans*, read depth greater than 50X provides good coverage of the genome and large contigs. As all of the current NGS technologies allow multiplexing, a 50X or lower depth of coverage provides opportunity for sequencing multiple samples per run thereby further reducing the cost of whole genome sequencing.
